# Supplementary material for: Rapid, Single-Step Protein Encapsulation via Flash NanoPrecipitation
Source: Polymers (Basel). 2019 Aug 27;11(9):1406. doi: 10.3390/polym11091406 (PMC6780228; doi:10.3390/polym11091406)
Supplement: Supplementary file 1 [file polymers-11-01406-s001.pdf]

polymers

MDPI

Supplementary Information

Rapid, Single-Step Protein Encapsulation via Flash NanoPrecipitation

Shani L. Levit, Rebecca C. Walker and Christina Tang*

Chemical and Life Science Engineering Department, Virginia Commonwealth University, Richmond, VA
* Correspondence: ctang2@vcu.edu

BSA Recovery

The BSA concentration in nanoparticles was measured with a Bradford assay. To prepare the nanoparticles for the assay, the nanoparticles were dissolved in Acetone, which precipitated the BSA. The samples were centrifuged and decanted. Followed by two more acetone washes. After the final decantation, the BSA precipitate was re-dissolved in water and the BSA concentration was measured with the Bradford assay. To determine the amount of BSA recovered after the acetone washes, a known amount of BSA was prepared in the same manner as the nanoparticles samples and the results from the Bradford assay were compared to the initial loading. We found that 98% of the BSA was recovered after the acetone washes.

Table S1. BSA recovery. BSA recovered in Bradford assay sample preparation.

Initial Loading (mg)	Recovered BSA (mg)	Percent Recovered (%)
0.90	0.88 ± 0.06	98% ± 3%

BSA-TA Precipitation

(a) BSA and TA were pipetted together at various ratios into deionized water (pH ~6).

(b) BSA and TA were pipetted together at a ratio of 9:5 by mass. The pH of the reservoir and the BSA solution were varied between pH 2-7. Images were taken 24 hrs after mixing when the precipitate settled.

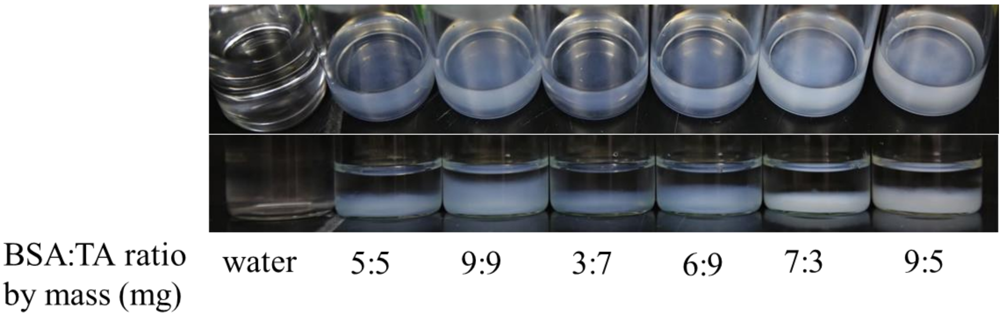

Figure S1a. Precipitation of BSA and TA - Varying BSA:TA ratio.

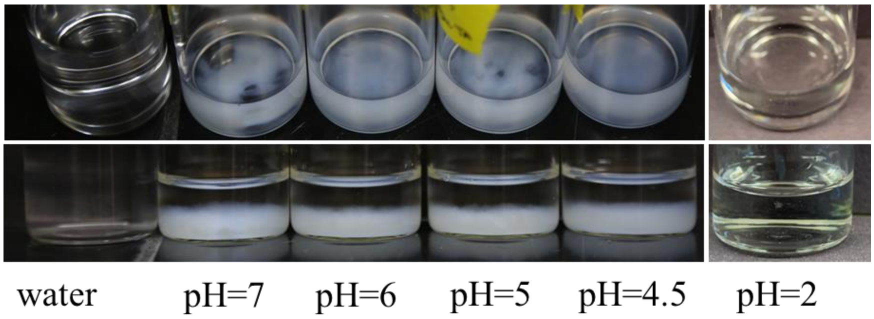

Figure S1b. Precipitation of BSA and TA – Varying pH.

PS-b-PEG, BSA, TA Nanoparticles

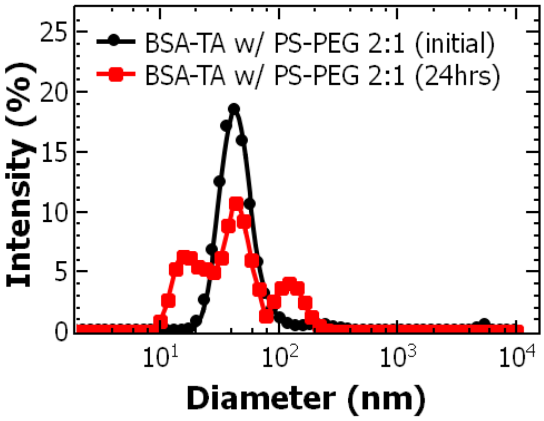

Figure S2. Nanoparticles formulated with PS-b-PEG (28 mg/mL) and TA (5mg/mL) dissolved in acetone and rapidly mixed with BSA (9mg/mL) dispersed in water via FNP.

PEI Properties

Table S2. Size and zeta potential of PEI dispersed in water.

Molecular Weight	Diameter (nm)	Zeta Potential (mV)
2kDa PEI	140 ± 99	13.4 ± 2.5
10kDa PEI	5.1 ± 0.7	12.3 ± 3
750kDa PEI	101 ± 7	18 ± 2.9

PEI, BSA, TA Nanoparticles

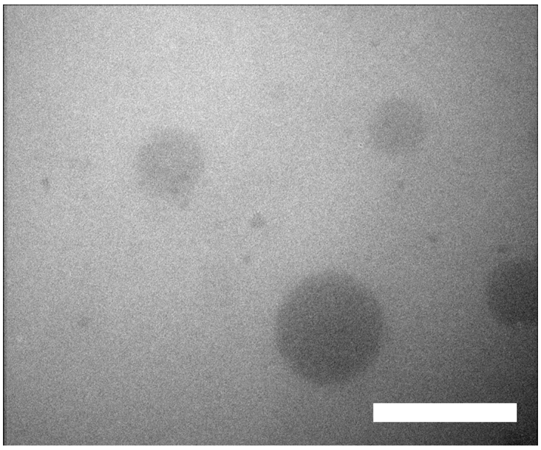

Figure S3. TEM of BSA/TA nanoparticles stabilized with 750kDa PEI with a 200 micron scale bar. The particles are spherical and the size is consistent with DLS measurements.

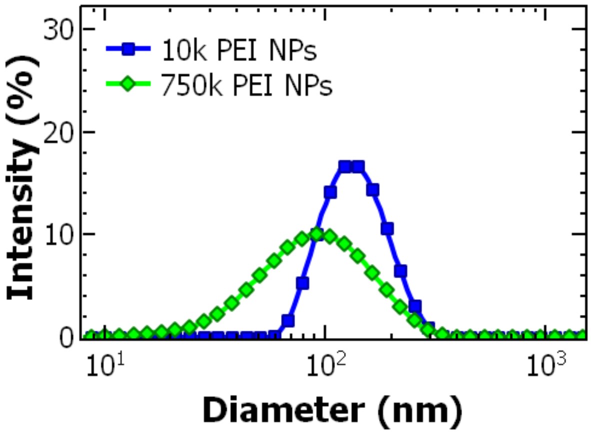

Figure S4. Effect of PEI molecular weight on nanoparticle formulation.

Table S3. Effect of PEI molecular weight on nanoparticle formulation.

Molecular Weight	Diameter (nm)	PDI	Zeta Potential (mV)
2kDa PEI	753 ± 230	0.596 ± 0.090	- 4.2 ± 1.1
10kDa PEI	153 ± 7	0.125 ± 0.022	+ 15.7 ± 1.0
750kDa PEI	101 ± 3	0.274 ± 0.007	+ 20.6 ± 1.8

Table S4. Effect of pH of the BSA stream and PEI reservoir on 750kDa PEI nanoparticles.

pH of BSA	pH of PEI	Zeta potential (mV)	Diameter (nm)	PDI
2	10	+ 23.2 ± 1.7	95 ± 8	0.340 ± 0.046
	2	+ 28.7 ± 17.9	115 ± 10	0.324 ± 0.100
5	10	+ 18.9 ± 0.9	105 ± 6	0.289 ± 0.017
	5	+ 38.7 ± 2.0	113 ± 8	0.304 ± 0.096
10	10	+ 24.0 ± 1.8	124 ± 13	0.434 ± 0.085

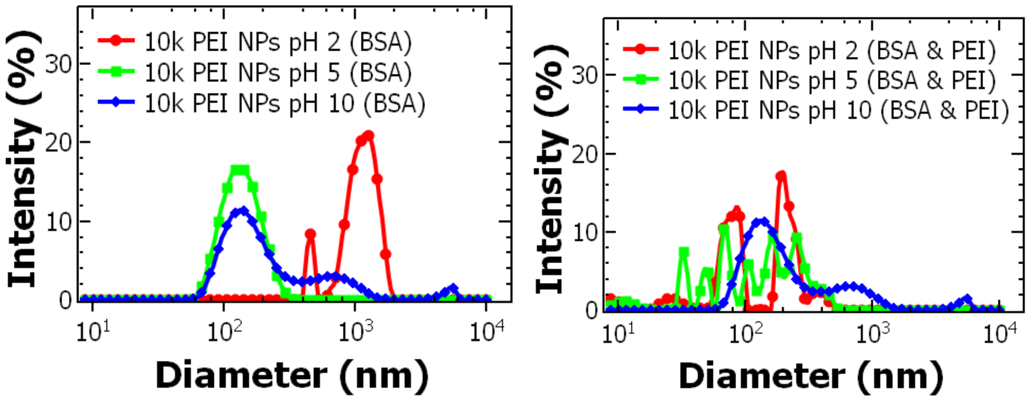

Figure S5. DLS of the 10kDa PEI nanoparticles formulated under different pH conditions by (A) varying the pH of the BSA stream and (B) varying the pH of both the BSA stream and the PEI reservoir. Stable particles were formed when the pH of the BSA stream was at or above 5 and the pH of the PEI stream was at or above 10. .

Table S5. Effect of pH of the BSA stream and PEI reservoir on 10kDa PEI nanoparticle formulation.

pH of BSA	pH of PEI	Zeta potential (mV)
2	10	+ 23.2 ± 1.7
	2	+ 31.3 ± 3.6
5	10	+ 15.6 ± 0.4
	5	+ 30.2 ± 2.2
10	10	+ 15.7 ± 1.1

Table S6a. Varying ratio of stabilizer to core for 750kDa PEI nanoparticles.

Polymer : Core	Initial		24 hrs	
	Size (nm)	PDI	Size (nm)	PDI
3 : 1	105 ± 2	0.279 ± 0.007	100 ± 3	0.260 ± 0.002
2 : 1	145 ± 39	0.442 ± 0.034	1257 ± 80	0.873 ± 0.109

Table S6b. Varying Total Solids of 750kDa PEI nanoparticles.

Total Solids (mg/mL)	Zeta Potential (mV)	Initial		24 hrs	
		Size (nm)	PDI	Size (nm)	PDI
2.8 mg/mL	+ 23.7 ± 1.8	107 ± 7	0.276 ± 0.005	107 ± 7	0.276 ± 0.005
5.6 mg/mL	+ 19.5 ± 1.2	99 ± 2	0.270 ± 0.009	98 ± 1	0.264 ± 0.006
11.2 mg/mL	+ 18.0 ± 0.4	101 ± 1	0.276 ± 0.009	101 ± 1	0.271 ± 0.005

Table S7. Varying total solids concentration and ratio of BSA to TA by mass for nanoparticles made with 10kDa PEI.

Polymer : Core	Total solids (mg/mL)	Size (nm)	PDI	Zeta Potential (mV)
3 : 1	5.6	143 ± 8	0.166 ± 0.033	+ 14.8 ± 1.1
3 : 1	11.2	319 ± 185	0.075 ± 0.051	+ 11.8 ± 1.0
2 : 1	4.2	136 ± 42	0.357 ± 0.055	+ 15.0 ± 1.4

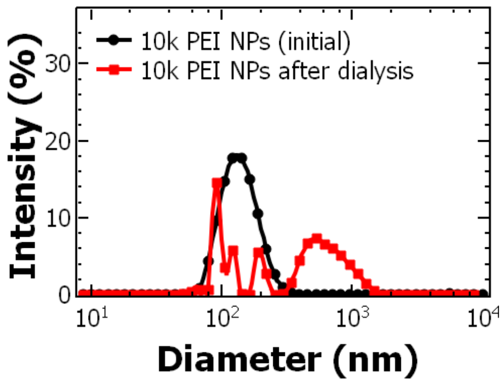

Figure S6. DLS of 10kDa PEI nanoparticles after dialysis.

Table S8. Effect of ionic strength on particle stability of 750kDa PEI nanoparticles.

Salt added	Concentration (mM)	Ionic Strength (M)	Diameter (nm)	PDI	Zeta Potential (mV)
Initial 750kDa PEI	0	0	107 ± 5	0.285 ± 0.004	18.5 ± 1.3
NaCl	10	0.01	89 ± 2	0.254 ± 0.007	12.7 ± 0.5
	30	0.03	87 ± 9	0.276 ± 0.039	15.8 ± 1.2
	100	0.1	91 ± 2	0.246 ± 0.004	14.8 ± 3.4
	300	0.3	84 ± 5	0.297 ± 0.032	20.0 ± 4.2
$CaCl_2$	10	0.03	84 ± 5	0.288 ± 0.028	23.1 ± 4.0
	100	0.3	85 ± 4	0.306 ± 0.0.024	21.2 ± 4.0

Table S9. Effect of TA on nanoparticle formulation.

PEI MW	Formulated with TA?	Size (nm)	PDI	Zeta Potential (mv)
10kDa	No	141 ± 13	0.196 ± 0.053	+ 16.1 ± 1.4
	Yes	153 ± 7	0.125 ± 0.022	+ 15.7 ± 1.0
750kDa	No	90 ± 11	0.301 ± 0.064	+ 23.5 ± 2.8
	Yes	101 ± 3	0.274 ± 0.007	+ 20.6 ± 1.8

© 2019 by the authors. Submitted for possible open access publication under the terms and conditions of the Creative Commons Attribution (CC BY) license (http://creativecommons.org/licenses/by/4.0/).
